# Using a theoretical framework to develop postgraduate health professions education research and practice

**DOI:** 10.15694/mep.2020.000078.1

**Published:** 2020-04-27

**Authors:** Derek Jones, Tim Fawns, Gillian Aitken

**Affiliations:** 1 University of Edinburgh

**Keywords:** Ecological systems theory, scholarship of learning and teaching, meta-framework, postgraduate education.

## Abstract

This article was migrated. The article was marked as recommended.

Healthcare education is complex and multifaceted, requiring study from different angles and with different lenses. We propose that the use of a meta-framework can help those teaching and researching postgraduate health professions education make holistic sense of their practice and findings from different projects. We discuss how we have employed Bronfenbrenner’s ecological systems theory (EST) as an overarching theoretical framework for the scholarship of learning and teaching in the context of postgraduate health professions education. Taking a structured approach to pedagogical thinking and research through the use of a meta-framework opens up useful ways of framing findings and further questions, locating research projects within a bigger picture, and communicating to others the focus of a research programme. We address the problem of the under-theorizing of educational research in postgraduate health professions education, advocating both theoretical frameworks for individual research projects, and an overarching theoretical “meta-framework” to interrogate and draw together multiple studies. In doing so we build on, critique and further develop Bronfenbrenner’s ecological systems theory.

## Using EST to develop a research programme

Recent years have seen a rapid expansion of postgraduate taught (PGT) programmes designed to enhance health professions education (HPE); many (including our own) are delivered online. The provision of continuing education for healthcare professionals is big business, as attested by the proliferation in the number of PGT-HPE courses offered worldwide, currently estimated at over 100 ( FAIMER, 2019). One institution alone reported 3438 graduates between the years 1988-2017 ( Sethi *et al.*, 2018). Drivers for the expansion of PGT-HPE include: regulatory changes requiring educators in the clinical setting to evidence and develop their teaching capabilities (e.g. General Medical Council, 2009); medical schools (and universities in general) responding to calls for the professionalization of teaching and enhancing the student experience ( Trowler, Fanghanel, and Wareham, 2005; Eitel, Kanz, and Tesche, 2000); and a suggestion that masters degrees are increasingly regarded as currency for career development ( Tekian and Harris, 2012).

PGT-HPE programmes are designed to develop clinicians who teach and provide academic credit for scholarship of teaching and learning (SoTL). However, the potential benefits are more far-reaching than the individual. Key to this argument is the recognition that such programmes offer an environment where healthcare and educational scholarship meet, providing a fluid interface between theory, practice, and research ( Ng, Baker, and Leslie, 2017). If qualifications in PGT-HPE do indeed have career progression currency, those completing masters degrees are increasingly likely to hold positions of responsibility and influence in a range of academic, clinical, and policy contexts.

Tobbell, O’Donnell, and Zammit (2010) stated almost a decade ago that the body of research addressing the experiences of PGT students in general is limited, a situation which continues. Given the increasing number of graduates and their potential impact on healthcare practice, it is surprising this area has not received more interest from the wider HPE community which, it has been suggested, has been orientated towards curricula, skills and attitudes, individual characteristics of medical students, and assessment of students and trainees ( Regehr, 2004). Some preliminary work on the experience of PGT-HPE suggests that graduates report increased self-efficacy for teaching and attest to the personally transformative nature of such education ( Sethi *et al.*, 2018). While this is encouraging, we also wish to consider the benefits of such programmes beyond those attributed to the particular characteristics of the individual.

This expansion of provision in PGT-HPE, and the limited research to date, provides an opportunity to engage with the PGT-HPE/educational research nexus at an early stage in its evolution. We believe this opportunity requires we conceptualise these programmes as providing an opportunity for discussion and sharing between the clinic and the academe ( Albert, Hodges, and Regehr, 2007; van Enk and Regehr, 2018), and moving from knowledge transmission to knowledge mobilisation ( Ng, Baker, and Leslie, 2017). As part of this project, we see locating research within a theoretical framework as providing the opportunity to explain, rather than simply describe, what is happening in a particular educational context. For some time it has been noted that the HPE literature tends to be lacking in a theoretical orientation and restricted to exploring what works rather than the mechanisms at work in producing relevant outcomes and shaping experiences ( Albert, Hodges, and Regehr, 2007; Cook, Bordage, and Schmidt, 2008; Laksov, Dornan, and Teunisson, 2017).

Adopting an overarching theoretical framework (or “meta-framework”) that can be applied across multiple studies - even where different methodologies have been used within those studies - complements a common practice in many University departments, which is to take a thematic (or programmatic) approach to research activity in order to develop a coherent body of work, build critical mass, increase research outputs and reputation. One of the problems in designing a research programme is the balance between taking funding opportunities as they arise whilst maintaining sufficient focus to have an impact and develop expertise. Using a unifying theory helps to connect a range of different projects. In the next section, we present Bronfenbrenner’s (1979) ecological systems theory (EST) and go on to explain how we have used it as a way of thinking about our programme and tying together a range of otherwise discrete research projects.

## A Theoretical Meta-Framework

The delivery of PGT programmes is complex and multi-faceted. As a team with responsibility for delivering a large online programme in Clinical Education we found it challenging to consider the numerous and interconnected factors likely to influence the student experience. Online PGT programmes at the University of Edinburgh are delivered on a part-time basis, thus allowing students to continue their clinical work whilst studying. As experienced professionals, students are encouraged to share and discuss their experiences with each other, so enriching the learning environment for all. Online masters degrees typically run over three years and allow students to build relationships, both with faculty and each other during this period ( Aitken, 2020). Students have a choice in where they wish to study, and it is important that a programme’s content is research informed and evidence based to ensure it remains competitive and viable.

In much of our research, we take an ecological view, locating the individual (and ourselves) within a wider environment. To this end, EST can be used as a theoretical meta-framework for understanding the interaction of different kinds of systems in which our PG students operate. EST was originally proposed in the context of child development and has since been used in a diverse range of health, educational, and other contexts (Costello and Donnellan, 1979; Onwuegbuzie, Collins, and Frels, 2013; Bluteau, Clouder, and Cureton, 2017; McLinden, 2017; Eriksson, Ghazinour, and Hammarström, 2018).

**Figure 1.**
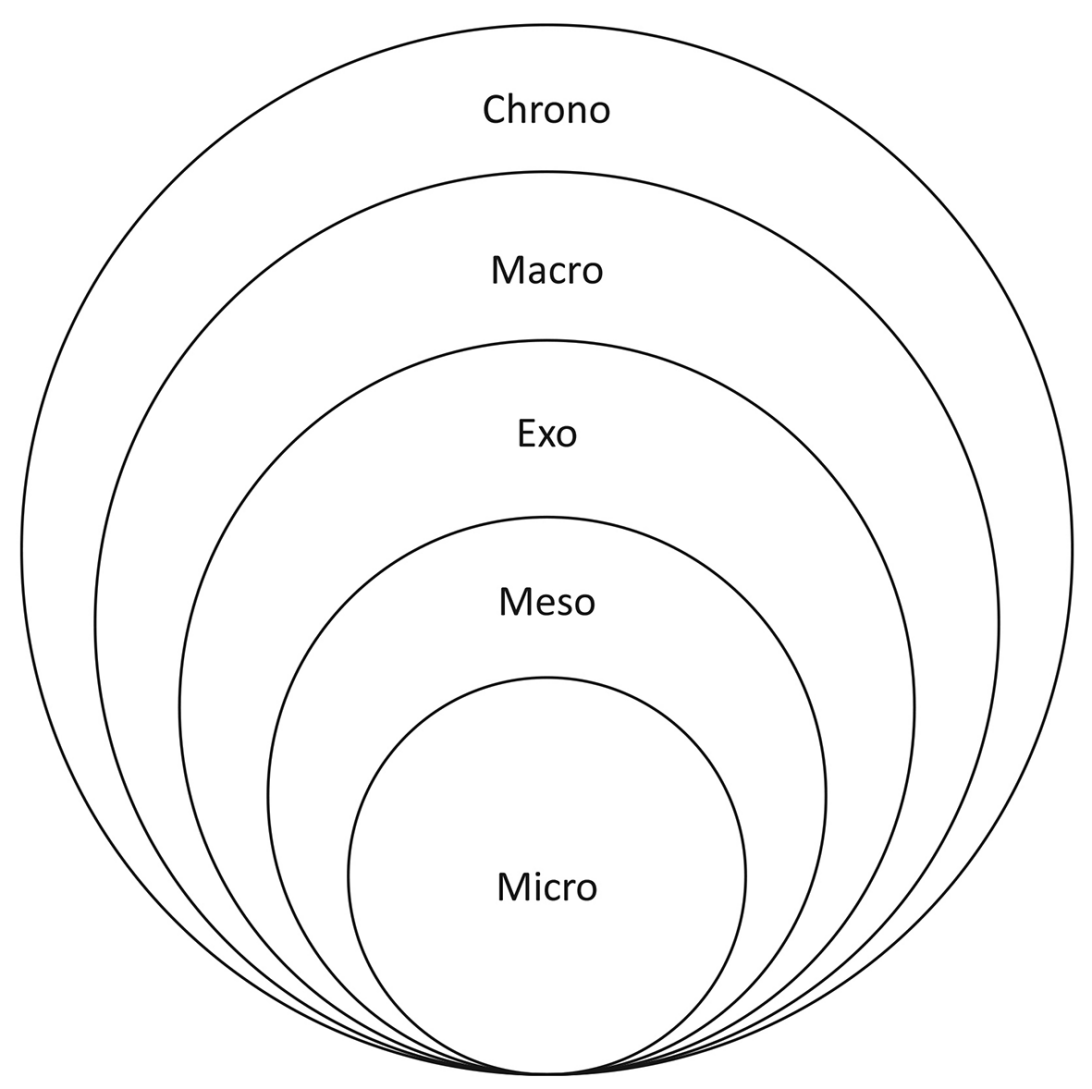
Bronfenbrenner’s Ecological Systems ( Bronfenbrenner, 1979; 1986)

Bronfenbrenner proposed that development is influenced by a set of ecological systems (micro, meso, exo, and macro) that operate at different distances from the child. These different systems represent the scope and immediacy of domains that influence the learning of the individual. For Bronfenbrenner, the microsystem was where the child was directly involved in social interactions and learning experiences (for example, the classroom). The mesosystem was comprised of connections between microsystems (for example, the home and classroom). Exosystems operated at a distance, influencing the individual but without their direct participation (e.g. government education departments). The macro-system was composed of societal features (e.g. cultural attitudes and beliefs). The chronosystem was added later to account for the change in systems over time ( Bronfenbrenner, 1986). Figure 1 shows Bronfenbrenner’s original conception of these systems in a nested arrangement.

**Figure 2.**
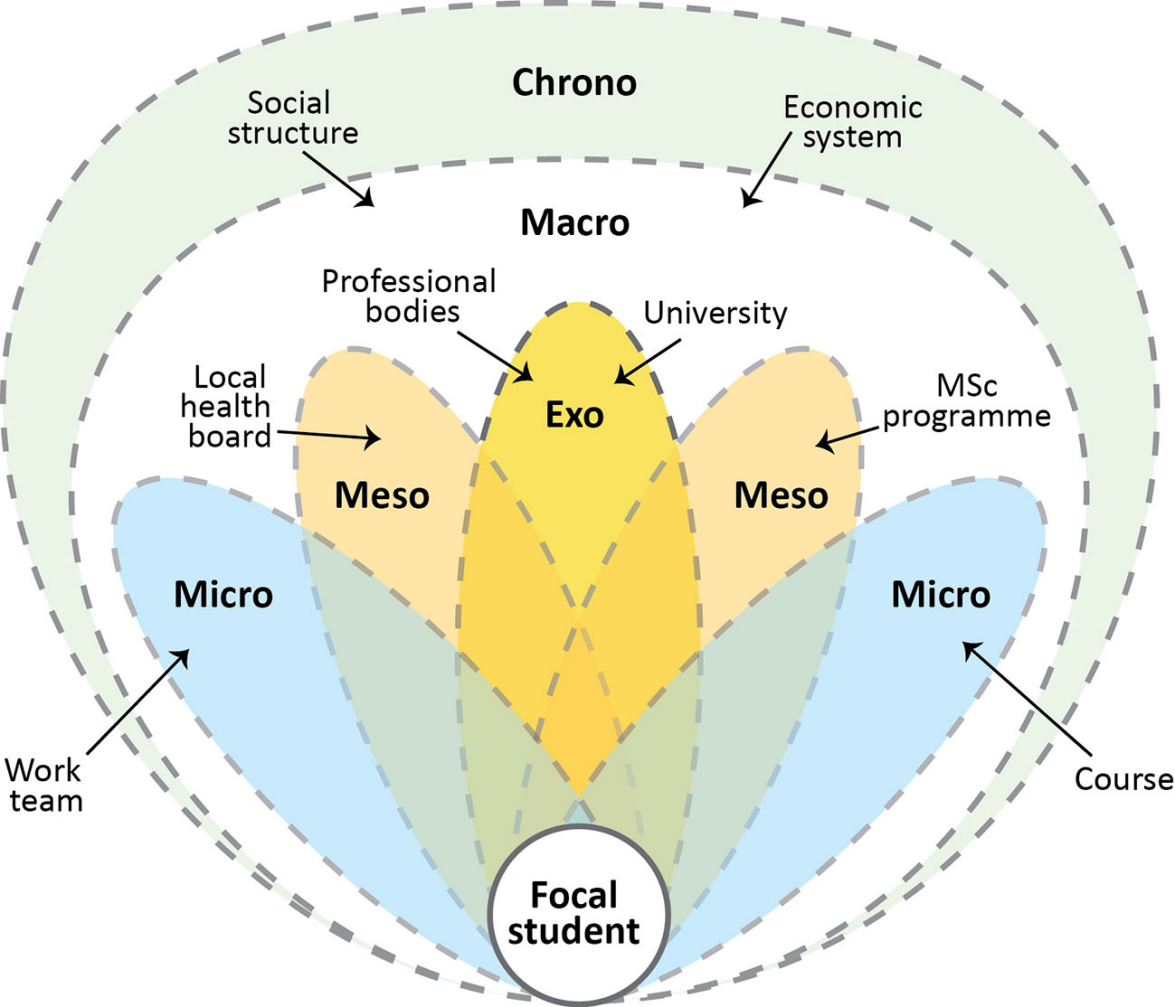
Ecological systems of an online postgraduate student

Neal and Neal (2013) have subsequently developed EST to better explain how each system relates to the others. Rather than each system being nested in the next (compare Figure 1 and Figure 2), Neal and Neal argued for a networked relationship between systems at different levels, meaning that microsystems (such as a course or professional team) are connected to, but not directly part of, mesosystems (such as an institution’s PGT community). Figure 2 expresses our conception of EST as it relates to one of our own PGT students, highlighting the complexity of the interactions. We use a flower head as a metaphor for the dynamic relationship between systems in which the student is at the centre and the petals represent multiple systems. The number of petals, their proximity to each other, and degree of overlap will vary from person to person. The chrono-system envelopes all of the petals and reflects the past, present and future of each of these systems, with some petals withering (but never completely disappearing) and new ones being added.

In the example given in Figure 2, the student (replacing Bronfenbrenner’s child) is positioned in the centre for the purposes of illustration, however ecologies do not have an absolute centre. Rather than always being at the centre, the student has a “relational position” within an ecosystem ( Elliott and Davis, 2018). Indeed, the flexibility to move the centre of our gaze is fundamental to our approach to researching HPE from different theoretical and conceptual perspectives. The number of elements, their proximity to each other, and degree of overlap will vary depending on who or what is in focus. The chrono-system envelopes all of the systems and reflects the past, present and future of each of these systems, with some fading and others coming into prominence.

This model can be used with a range of social and educational theories to explore many aspects of PGT-HPE programmes and their wider relevance. It can be used to consider different contextual levels in which interactions occur and generate researchable questions, for example:

• interactions at the level of a course (micro-system);
  • e.g. exploring the relationship between dissertation supervisor and supervisee ( Aitken *et al.*, 2020);
• locating individual students (and their learning) within interconnected systems (meso- and chrono-system);
  • e.g. exploring the links between students experiences on PGT-HPE programmes and their impact in the workplace, and following this up over time ( Aitken, 2020);
• critically reflecting on goals, design, scope, and potential impacts of PGT-HPE (macro-system);
  • e.g. exploring conceptualisations of evaluation of teaching and ideologies underpinning proposed outcomes of education ( Fawns, Aitken, and Jones (2020);
  • the policy and practice context of faculty development (exo-system);
  • e.g. analysing the operationalisation and impact of faculty development policy and practice documents (in progress).

Using EST as a meta-framework, in combination with study-specific theories (such as Bourdieu’s [1986] concept of habitus), can overcome a potential criticism that its broad scope makes it difficult to use as the basis for addressing specific questions. In the next section, we expand on the examples above to show how we have used EST to compare, contrast, and synthesise the findings of a selection of research projects we have undertaken, and how EST has helped us to understand a variety of phenomena pertinent to each system level, and the relationships within systems as well as across them.

## Using EST to support research, scholarship and pedagogical thinking

At the micro level, individual students on the MSc Clinical Education develop their understanding of teaching and learning during their programme of study, as well as obtaining academic credit. Many find their conceptions of teaching shift as they develop their ability to engage in the discourses of education and take a more active focus on the needs of the learner and the aims of their own teaching. At the same time, an EST lens allows us to move beyond considering the development of an individual and towards considering PGT-HPE as a distributed phenomenon that crosses the boundaries of systems and is affected by multiple layers (Tobell, O’Donnell, and Zammit, 2010). Throughout their studies and, crucially, after graduation, our students engage with the wider community to a greater or lesser degree. Graduates take their learning back to their own areas of practice (meso-level), and can, in some cases, mobilise their new knowledge to impact on the macro and exo levels. Thus, alongside developing their own practice in clinical settings, there is a suggestion that those completing masters degrees are becoming the new leaders in the field ( Tekian and Harris, 2012), and can increasingly contribute to, and frame, academic debate. Therefore, thinking of online PGT programmes solely in terms of microsystems and mesosystems would risk losing sight of the potential impact and reach of such programmes.

Drawing on our own experience, an illustrative example of such influence would be students from Poland learning about the system of appraisal in the UK as part of their studies. This learning is taken back to the individual’s hospital or university and considered in her or his own context, thus influencing the macro culture of medical education in Poland. Locating this activity within a network of ecological systems helps us to explore the impact of our postgraduate, online programme, and illustrates why the wider educational community, in clinical and university settings, should be interested in what is being taught and its practical consequences.

PG students are at the centre of their own circle (micro-level), but also on the periphery of those of others’ (meso-level) (see Fig 2). Key to the development of the student in the academic mesosystem is the interaction with both the academic tutor and the wider student body. It is a specific aim of PGT to develop the agency, self-regulation and critical thinking of PGT students ( Quality Assurance Agency for Higher Education, 2013), and so such programmes are designed to encourage active engagement and participation. Those choosing to undertake a postgraduate degree in healthcare education are not only learning to develop skills in contributing to, and critiquing, academic discourse but have the additional challenge of engaging with the unfamiliar terminology used in educational research. At the same time, our EST meta-framework helps us understand how these things are relational and contextual; they are ongoing accomplishments that must be maintained within and across settings and systems. This highlights the need for the development of higher-level skills or attributes, and sustainable capacities for learning and adapting.

At the exo level, we are conscious that PGT-HPE (at any level) does not sit outside of its political and socio-economic context and boundaries between institutions of higher education, workplaces, and civic society are increasingly permeable ( Boud and Rooney, 2015). We explicitly seek to explore these connections in all of our courses and draw on the international experiences of our students. We have examples of our graduates influencing the “policy-making community” in their context and, indeed, it is a reasonable aim of many PGT programmes that their graduates aspire to exercising influence at this level. This highlights that the exo-system has to be conceptualised more in line with the suggestion of Neal and Neal (2013) who saw the systems as being interacting and open rather than distinct. We see it as part of our role to reflect on, and break down, the distinctions between systems to facilitate the development of our students’ agency across different spheres of influence. We see the EST framework as helpful in recognising the increasing influence of PGT graduates, rather than just seeing them as people going through a process of being educated. For us, PGT students have increasing outward influence on the different systems, potentially all the way out to the macro-system.

Considering the chrono-level system has caused us to reflect on the temporal dimensions of education. Not only do we want to develop student agency across settings, but also in relation to adapting to, and integrating into, shifting contextual landscapes. We aspire to produce graduates who are able to negotiate changes in policy, technology, culture, economics and politics such that they not only maintain but also enhance their own agency and authority. Thus, considering the chrono-system helps us to think about the importance of adapting to dynamic, rather than static, contexts at each level (micro, meso, macro, exo), particularly in times of political and economic uncertainty. The current COVID-19 crisis further emphasises how the context of health professions education can change rapidly in a short space of time.

## Using EST to develop a research programme

In response to the issues noted above, as a programme team we have been working to develop a theoretically informed, coherent programme of research. To this end, we began by compiling a list of theories (or concepts) that we have found ourselves returning to in our scholarly discussions. This includes (but is not limited to) the work of Bourdieu (1986), and Derrida (2000); and concepts of distributed learning ( Sutton, 2010), sociomaterialism ( Fenwick, 2015), and practice theory ( Schatzki, 2001). We were mindful of Ng, Baker, and Leslie’s (2017) proposition that healthcare professionals move between settings (classrooms, hospitals, communities) that are dynamic, as the people, practices, and technologies and materials within them change. Further, each of these settings is implicated in wider systems that include clinical practice, clinical education, healthcare systems, policy, and the wider society. We agree with Ellaway, Bates, and Teunissen (2017) that context should not be treated as an interfering variable but as a fundamental component. Indeed, it is not only context, but also contextual change that we need to address in the education of health professionals. With this in mind, we wanted to be able to evolve a programme of research that allows us to take advantage of opportunities to conduct (in some cases) small-scale projects that would still have a connection to a larger whole made explicit via EST.

Starting at the micro level, we reflected that individual experiences would be constructed by direct experience of interactions with other students, technology, tutors, colleagues and family. Research at this level could be directed at exploring individuals’ backgrounds, motivations for PG-HPE and benefits gained. This takes account of our experience that students are a diverse group in terms of age, sex, professional background, career point, and culture. Reasons for engaging in study vary but initial work we have undertaken suggests such vocationally related programmes offer potential career advantages in terms of promotion and recognition. Knowledge gained from investigation at this level is valuable in its own right in terms of understanding our learners and the graduate attributes they acquire. However, the application of an overarching theoretical framework allows for moving beyond an individualistic approach. For example, our group has been exploring graduate attributes, looking at this in the context of the EST prompted us to think about this topic in terms of the wider organisational and social system using Bourdieu’s concepts of field, habitus, and capital ( Aitken *et al.*, 2019).

Whereas research at the micro level may be orientated towards individual agency (or perhaps distributed or relational agency within an immediate setting) considering the mesosystem allows a focus on the influence of structure. We could, for example, explore postgraduate programmes in terms of the ways in which participants’ experiences and outcomes are shaped by the design of the curriculum. Knowledge gained at this level has potential value in terms of evaluating the curriculum in relation to its purpose. An important consideration is how such programmes are designed and delivered in practice; linking the programme as structure and the team as individuals. Our point is that one area cannot be viewed in isolation

The exo-system was considered by Bronfenbrenner to be out of reach of the individual, who was assumed not to have an impact on decision making at this level. An example of the exo-system impacting PGT students is the policy decisions by regulatory bodies, for example in the UK the General Medical Council (2009) and General Dental Council’s (2018) requirements to acquire evidence of necessary skills as a trainer. Research focused at this level could usefully explore how institutional processes support (or hinder) learning. Using EST reminds us that the link between structure and agency is interactive rather than deterministic. In the spirit of Marx (1957), people construct their learning but not in circumstances of their own choosing. Although at the time of their studies the individual student may have no impact on decision-making, our experience is this situation may evolve as the student becomes more senior and more comfortable in engaging in academic debate, thereby influencing those across different ecological systems including institutional and regulatory bodies. Furthermore, because most participants remain engaged in professional practice alongside their studies, they may become increasingly active participants in other, practice-based mesosystems. Participation in PGT-HPE affords the possibility of enhanced, informed, dialogue between immediate work teams and wider clinical departments or institutions.

The literature on graduate attributes highlights the apparently transferable skills that graduates acquire on completion of their studies ( Barrie, 2012) resulting in capacity to mobilize knowledge, thus meeting the challenge described by Albert, Hodges, and Regehr (2007) of balancing science and service. Our current research explores some of the challenges and the EST framework helps us think through how easily (or otherwise) transferability happens in reality. EST also provides a framework for exploring the longer-term impact of PGT-HPE, investigating the influence of graduates on exosystems and even macrosystems over time. It also helps us to understand how graduates negotiate the requirement to adapt to and integrate into different ecosystems. Such research could make explicit for employers the value of investing in students participating in PGT-HPE.

Consideration of the macro-system leads to exploration of the interaction between socio-cultural context and HPE. It provokes questions around how we as a society view and value education, and how this is embodied in policies, the design of programmes, and student engagement in those programmes. EST encourages us to think in broad theoretical terms about PGT-HPE and highlights the value of, for example, critical realism, evident in the work of one of our PhD candidates who has used this approach to explore why clinical educators feel they are not valued. Such approaches explicitly investigate the ecological system as it applies to PGT-HPE as a whole.

In the context of innovation research, Costello and Donnellan (2012) highlighted the potential of EST in explaining how innovations develop, grow and decay. Similarly, research in PGT-HPE can usefully explore, at the chrono-system level, changes in learning and teaching practices and impact over time. An example would be our work with programme graduates looking at their development over time, within and across ecological systems.

## Conclusion

We have presented an example of how an overarching theoretical framework can help educators make sense of a range of teaching and research initiatives. We have offered ecological systems theory as an example of a potentially-valuable theoretical meta-framework that has enabled us to respond to the need for theoretically informed research into PGT-HPE that locates the individual student within a wider (including temporal) context that involves interactions within and between different systems. We have found that EST can assist us in the process of integrating our critical reflection on our teaching and learning activities with developing a research programme. Using a framework to structure our thinking has enhanced our scholarly discussions about the characteristics, aims and impact of PGT-HPE. As others have found, there is no reason why some frameworks, such as EST, cannot be used for a wide range of purposes, including any kind of educational research to help scholars think about their particular topic and develop questions - whether that be PBL, widening access, professionalism, or teamwork to name but a few.

## Take Home Messages

This paper addresses an important topic in health professional education -- that is, the use and development of a meta-theoretical framework that assists in describing and positioning the findings of diverse studies and reflecting on practice.

## Notes On Contributors

Derek Jones

Derek is the Programme Director of the PhD Clinical Education programme at the University of Edinburgh. ORCiD: https://orcid.org/0000-0003-2197-7657

Tim Fawns

Tim Fawns is the Deputy Programme Director of the MSc Clincial Education programme at the University of Edinburgh. ORCiD: https://orcid.org/0000-0001-5014-2662

Gillian Aitken

Gill Aitken is the Programme Director of the MSc Clinical Education programme at the University of Edinburgh. ORCiD: https://orcid.org/0000-0002-5492-1943
