# Supplementary material for: Using a theoretical framework to develop postgraduate health professions education research and practice
Source: MedEdPublish (2016). 2020 Apr 27;9:78. [Version 1] doi: 10.15694/mep.2020.000078.1 (PMC10697538; doi:10.15694/mep.2020.000078.1)
Supplement: Peer review history

# Reviewer report: Reviewer response for version 1

**DOI:** 10.21956/mep.20049.r30724

**Published:** 2020-05-14

**Authors:** Ngusha Anyam

**Affiliations:** 1 University of Ibadan

This review has been migrated. The reviewer awarded 4 stars out of 5 A paper that emphasizes the importance of using a framework to structure research in postgraduate health professions education, that would reflect on practice. It is an informative and interesting article which provides a repository of valuable resources for research.

Reviewer Expertise:

NA

No decision status is available

# Reviewer report: Reviewer response for version 1

**DOI:** 10.21956/mep.20049.r30721

**Published:** 2020-05-01

**Authors:** Cesar Orsini

**Affiliations:** 1 UEA, Norwich Medical School.

This review has been migrated. The reviewer awarded 4 stars out of 5 I thank the authors for submitting this manuscript. I enjoyed reading and learning from their discussion on Bronfenbrenner's ecological systems theory as a theoretical framework for the teaching and research in postgraduate health professions education.I agree with the authors claiming that healthcare education is complex and multifaceted and that different perspectives are needed. Some (or most)of these come from the social sciences and represent an area that we, having a clinical background, are not so familiar with. In this sense, this paper is valuable and contributes to the discussion of using different/innovative perspectives and theoretical frameworks in health professions education.The first part of the manuscript, under the heading "Using EST to develop a research programme", presents a well-written introduction and justification. However, I was expecting this section to be more informative and explicit about EST.Figure 2 and the explanation of the authors to Bronfenbrenner's ecological systems theory applied to their student in their online course is informative and clearly illustrates the point on how this model can be applied to HPE research. As I read through, I found the section on "Using EST to develop a research programme" to be particularly relevant to allow the readers to transfer the authors' ideas to their own research settings.This paper provides a valuable resource for researchers as Bronfenbrenner's EST may be an applicable underpinning theory and provide structure to a vast range of studies conducted in HPE, whether it may be in an under or postgraduate context.

Reviewer Expertise:

NA

No decision status is available

# Reviewer report: Reviewer response for version 1

**DOI:** 10.21956/mep.20049.r30723

**Published:** 2020-05-01

**Authors:** Reyam Faisal

**Affiliations:** 1 Maternity hospital

This review has been migrated. The reviewer awarded 5 stars out of 5 really interesting article opens the mind in thinking differently regarding the thorough knowledge that starts from student till echo system and social structure

Reviewer Expertise:

NA

No decision status is available

# Reviewer report: Reviewer response for version 1

**DOI:** 10.21956/mep.20049.r30722

**Published:** 2020-04-29

**Authors:** Balakrishnan Nair

**Affiliations:** 1 Centre for Medical Professional Development and University of Newcastle

This review has been migrated. The reviewer awarded 4 stars out of 5 This paper highlights an exciting and innovative way of looking at Postgraduate education. Any education needs to be transformative. Particularly in the postgraduate HPE field ,it should have positive impact on the health care system , patients and the population. This model brings the individual learner and all other components together. I was able to use this model on a leadership program I was running and reflected on the various outcomes and found this way of thinking very useful when setting up new courses.

Reviewer Expertise:

NA

No decision status is available

# Reviewer report: Reviewer response for version 1

**DOI:** 10.21956/mep.20049.r30726

**Published:** 2020-04-28

**Authors:** Samar Ahmed

**Affiliations:** 1 Ain Shams University Faculty of Medicine

This review has been migrated. The reviewer awarded 4 stars out of 5 This work is extraordinary in my point if view. It offers a reflection in the use of an already existing theory of the ecological system effect on learners to develop a program. This is extremely useful and replicable and has implications on program reform towards establishing a true educational impact on PG students.

Reviewer Expertise:

NA

No decision status is available

# Reviewer report: Reviewer response for version 1

**DOI:** 10.21956/mep.20049.r30725

**Published:** 2020-04-27

**Authors:** Felix Silwimba

**Affiliations:** 1 University of Lusaka

This review has been migrated. The reviewer awarded 5 stars out of 5 informative ,educative and informs the growing importance of medical education.

Reviewer Expertise:

NA

No decision status is available
